# Investigating the establishment of the rumen and oral bacterial communities in beef cattle and assessing the applicability of using the oral bacterial community composition as a proxy for rumen bacterial community structure in cattle

**DOI:** 10.3389/fmicb.2025.1667498

**Published:** 2025-12-19

**Authors:** Seidu Adams, Andrew Lakamp, Nirosh Aluthge, Emma Laible, John Dustin Loy, Matthew L. Spangler, Samodha C. Fernando

**Affiliations:** 1 Department of Animal Science, University of Nebraska-Lincoln, Lincoln, NE, United States; 2 School of Veterinary Medicine and Biomedical Sciences, University of Nebraska-Lincoln, Lincoln, NE, United States

**Keywords:** oral bacterial community, 16S rRNA gene, rumen bacterial community, cattle, host phenotypes, microbiome

## Abstract

**Introduction:**

Studies have investigated the rumen microbiome composition and functions to improve ruminant agriculture and its environmental impacts. Yet, sample collection for rumen microbiome analysis can be difficult and invasive, hindering the ability to sample large animal populations. Studies have proposed using oral swabs as an alternative to rumen sample collection. Here, we investigated the potential of using the oral bacterial community as a proxy for the rumen bacterial community during the cattle production cycle.

**Methods:**

We investigated the development of the bovine rumen and oral bacterial communities using longitudinal sampling and the applicability of using the oral to predict host phenotypes. To this end, we utilized 16S rRNA gene sequencing to characterize and compare the rumen and oral bacterial community composition over multiple time points using amplicon sequence variants (ASVs) in a beef cattle population of 166 animals. Additionally, host phenotype of weaning weight was predicted using the Bayesian ridge regression model to evaluate the applicability of using the oral bacterial community for phenotype prediction.

**Results:**

Our results identified the rumen and oral bacterial communities to have different trajectories of assembly. The proportion of *Proteobacteria* and *Actinobacteriota* was higher (*p* < 0.0001) in the oral samples. Whereas rumen samples had greater abundance of members of the phyla *Bacteroidota*, *Firmicutes*, *Verrucomicrobiota*, *Fibrobacterota*, and *Spirochaetota*. The investigation of the oral and rumen bacterial community establishment demonstrated considerable dynamism, where diet and age-related factors to contribute toward bacterial colonization through introduction of new species and the proliferation of early colonizers. Finally, a Bayesian ridge regression model was developed to estimate weaning weight using the centered and scaled log-transformed relative abundance of ASVs. The proportion of variation explained in weaning weight by the oral and rumen bacterial communities were 30 and 37%, respectively.

**Discussion:**

Results from this study suggest that oral and rumen bacterial communities are distinctive, and the oral bacterial community may not serve as a good proxy for the rumen bacterial community even in adult animals with a well-established microbiome. However, the oral bacterial community may serve as a proxy for phenotypic traits of interest in beef cattle.

## 1 Introduction

The rumen harbors a diverse and complex microbial community (Malmuthuge and Guan, 2017), where diet has been shown to greatly influence the rumen microbial community composition and function (Fernando et al., 2010; Abbas et al., 2020; Anderson and Fernando, 2021). In addition to diet, rumen microbial community composition and function changes as the animal ages (Sha et al., 2020; Hao et al., 2021; Yin et al., 2021). Specifically, until 60–90 days of age, the calf is considered a pre-ruminant and during this time the rumen is not fully functional (Govil et al., 2017). The composition and function of this mature complex microbial community has been shown to be associated with traits such as feed efficiency (Shabat et al., 2016; Li and Guan, 2017), methane emission (Wallace et al., 2015; Difford et al., 2018), milk yield and milk composition (Jami et al., 2014), and disease states (McCann et al., 2016). Many of these studies have utilized small animal numbers to investigate the rumen microbiome due to the difficulty and invasiveness of current rumen sample collection methods (Huws et al., 2018; Fortina et al., 2022; Hagey et al., 2022). However, large-scale animal studies investigating the rumen microbiome and production phenotypes are needed to develop effective microbiome manipulation strategies to improve ruminant health and productivity (Tapio et al., 2016). The current gold standard for rumen sample collection involves animal cannulation which requires specialized facilities for surgery rendering it impractical for large-scale sampling efforts. Additionally, cannulation reduces the value of the carcass for sale, limiting the involvement of producers in research studies (Kristensen et al., 2010; Marques et al., 2014; Ghazy, 2017). As an alternative to cannulation, Paz et al. described esophageal tubing and demonstrated esophageal tubing to provide a representative sample from the rumen similar to canula sampling using 16S rDNA sequencing (Paz et al., 2016). Although this approach is less invasive and less costly with no damage to the carcass, it is still unpleasant for the animal, can be contaminated with saliva, and may affect ruminal fermentation (Shen et al., 2012). Therefore, leveraging the physiological behavior of regurgitating rumen content for further mastication in ruminants (Kennedy, 1985; McLeod and Minson, 1988), recent studies have proposed oral swabbing as a non-invasive sampling approach as an alternative to esophageal tubing and traditional rumen sampling through cannulation (Kittelmann et al., 2015; Tapio et al., 2016; Young et al., 2020; Amin et al., 2021; Miura et al., 2022). Small-scale studies using oral swab samples have reported the oral microbiome to be a proxy for feed intake in dairy cows (Marcos et al., 2024) and have suggested the oral bacterial community as a proxy for the rumen bacterial community (Kittelmann et al., 2015; Tapio et al., 2016; Amin et al., 2021). These studies have observed strong correlations between oral and rumen bacterial communities, suggesting that oral swabs could predict rumen microbial community structure and composition (Tapio et al., 2016). Additionally, Kittelmann et al. successfully recovered a substantial proportion of rumen bacterial, archaeal, ciliate protozoa, and anaerobic fungal taxa in oral swab samples, where oral bacteria such as *Actinobacillus*, *Bibersteinia*, *Fusobacterium*, *Haemophilus*, *Mannheimia*, *Moraxella*, and *Neisseria* were the most abundant (Kittelmann et al., 2015). Studies comparing the oral and rumen bacterial community composition have utilized small sample sizes and have compared bacterial communities at higher taxonomic levels such as family and phylum levels (Kittelmann et al., 2015; Tapio et al., 2016; Amin et al., 2021). Many different bacterial species and strains can belong to the same higher taxonomic levels such as family and phylum. As such, whether the oral bacterial community can be used as a proxy for rumen bacterial community composition and function remains unclear. In this study, we investigated early rumen and oral bacterial community assembly and compositional dynamics longitudinally at ASV level resolution to identify if the oral bacterial community composition can be used as a proxy for rumen bacterial community composition. Additionally, studies investigating rumen bacterial community establishment in cattle with longitudinal sampling has been limited to dairy cattle (Jami et al., 2013; Huuki et al., 2022), with distinct differences in rumen bacterial community composition in dairy and beef cattle, here we describe bacterial community establishment in beef calves. Finally, due to the ease of oral microbiome sampling, we investigated the potential of using the oral bacterial community composition to predict weaning weight.

## 2 Materials and methods

### 2.1 Ethics statement

This study was approved by the University of Nebraska-Lincoln Institutional Animal Care and Use Committee under project ID 2720.

### 2.2 Animal management

**Figure 1:**
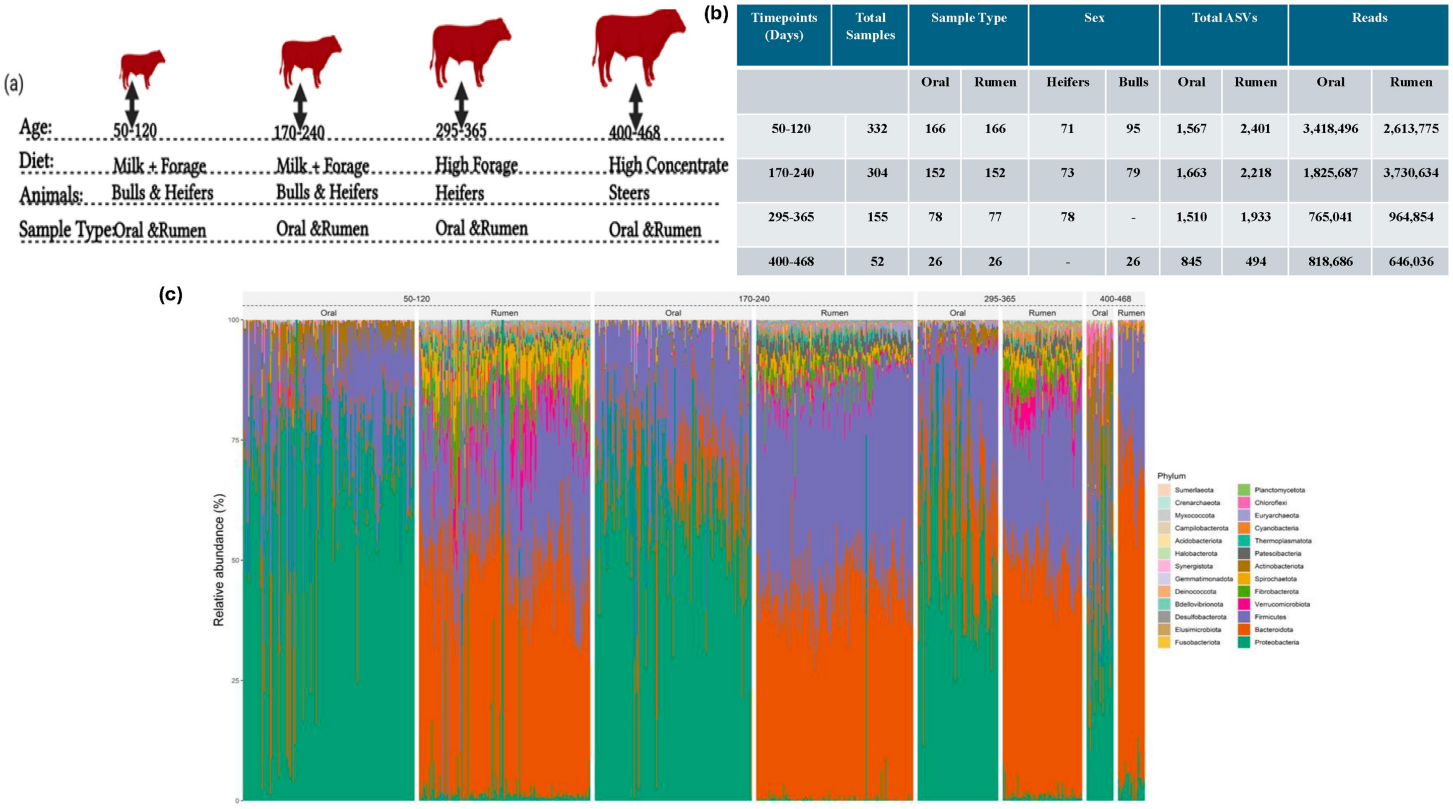
Overview of the experimental setup: **(a)** Animal numbers and diet information and sample types collected at each time point; **(b)** Table displaying read depth, sample types, and ASVs identified across time points; **(c)** Phylum level stacked bar plot of oral and rumen microbiome composition at different age groups.

We utilized the University of Nebraska teaching herd for this study which included 166 animals. The beef cattle population (*n* = 166) was longitudinally sampled at four distinct time points, at 50–120 days of age (the youngest animal was 50 days of age and the oldest was 120 days of age; both heifers and bulls), 170–240 days of age (the youngest animal was 170 days of age and the oldest was 240 days of age; both heifers and bulls), 295–365 days of age (the youngest animal was 295 days of age and the oldest was 365 days of age; all heifers), and 400–468 days of age (the youngest animal was 400 days of age and the oldest was 468 days of age; all steers) as illustrated in Figures 1a,b. All animals were subjected to the same management strategy until weaning and after weaning the heifers and steers were managed differently. The animals were subjected to three dietary regimens which included diet A (milk + forage diet - both heifers and bulls), diet B (high forage diet - heifers after weaning), and diet C (high-concentrate diet - steers after weaning). All animals were managed and allowed to graze in the same pastures and were kept with the dams until weaning at age 170–240 days old at which point weaning weights were collected. All animals had ad-libitum access to feed and therefore should not have limited regurgitation before sampling effecting oral microbiome sampling. Post-weaning, the animals were grouped by sex. Some heifers were retained in the herd for breeding and were fed a high-forage diet (diet B), while the bulls were castrated, moved to the feedlot, and fed a high-concentrate diet (diet C). For the steers, feedlot samples were collected just before feeding as suggested in a previous study for oral sample collection (Young et al., 2020). Animals collected at each time point, sample type, and diet information are shown in Figure 1b. Animals were weighed at weaning and weaning weight was collected. Both rumen and oral samples were collected from each animal at each sampling time point.

### 2.3 Sample collection

All rumen samples at all different age groups were collected via esophageal tubing, as previously described (Paz et al., 2016) to represent total rumen contents (rumen fluid and particles). Briefly, a Frick speculum was used to gain access to the rumen and the stomach tube (0.375″ internal diameter tubing with holes in the last 6″ of the tube to act as a strainer) was inserted and the rumen samples was collected using a vacuum pump. In addition to getting rumen fluid and feed particles smaller than 0.375″, particles trapped in the holes and the mouth of the tube was also added to the sample. Therefore, the collected samples comprised both rumen fluid and fibrous particulate matter and represents total rumen contents (solid and liquid fractions) and were used for microbial community analysis. Oral samples were collected before rumen sample collection using cotton tipped swabs (sterile cotton-tipped applicator, Puritan Medical Products Company LLC) (Tapio et al., 2016; Young et al., 2020). Briefly, the animals were restrained in the chute and a sterile cotton tipped swab was used to collect a combination of oral fluids and particulate rumen contents by swabbing the total oral cavity including sides, tongue and under the tongue. We expected rumen fluid to be present under the tongue as most rumen fluid is swallowed back. The presence of rumen particulate matter during collection suggested recent regurgitation with respect to sampling time. By ensuring the collection of rumen particulate matter during oral sample collection when available, we are confident that we took all possible attempts to investigate both particle and fluid associated rumen microbes from the oral samples. The samples were snap-frozen on dry ice and transported to the laboratory. Upon reaching the laboratory, samples were stored at −80 °C until used for bacterial community analysis.

### 2.4 DNA extraction, library preparation and sequencing

Total DNA extraction of the rumen and oral samples were performed using the Mag-Bind® Stool DNA 96 kit (Omega Bio-tek, Inc., Norcross, GA) according to the manufacturer’s protocol with a few modifications (Paz et al., 2018). The changes included the bead-beating of the samples at a frequency of 20 Hz for 10 min using the TissueLyser (Qiagen, Germantown, MD), the addition of RNase A (NEB, Ipswich, MA) to the lysis solution to ensure the removal of RNA and incubation for 10 min at 90 °C in a water bath between beat beating steps to further enhance bacterial cell lysis using temperature. Negative control DNA extractions were also performed to identify kit contaminations. Following DNA extraction, DNA was visualized on a 1.5% agarose gel using gel electrophoresis to assess quality of DNA extracted. The resulting DNA was used for 16S library preparation as described previously (Kozich et al., 2013). PCR amplifications were performed using primers targeting the V4 hypervariable region of the 16S rRNA gene with barcodes and adaptors to bind to the Illumina flow cell added to the 5′ end of the primer as described previously (Kozich et al., 2013). Each sample was amplified using uniquely barcoded dual index primers. The 25 μL PCR consisted of 1X Terra™ PCR Direct Buffer (Takara Bio Inc., Mountain View, CA), 0.625 units of Terra™ PCR Direct Polymerase (Takara Bio, Inc., Mountain View, CA), 2.5 μM of barcoded primers, and 20–50 ng of oral or rumen DNA. The cycling conditions consisted of an initial denaturation step at 98 °C for 3 min, followed by 25 cycles of 98 °C for 30 s, 55 °C for 30 s, and 68 °C for 48 s, with a final extension at 68 °C for 4 min (Paz et al., 2018). Following amplification, the PCR products were visualized on a 1.5% agarose gel for correct fragment size and were normalized using the NGS Normalization 96-Well Kit (Norgen Biotek Corp., Thorold, ON, Canada) according to the manufacturer’s protocol, with the modification of an additional 5-min spin before the final elution step. The normalized libraries were pooled and further purified and concentrated using the NucleoSpin® Gel and PCR Cleanup kit (Macherey-Nagel, Düren, Germany), as described by the manufacturer. The resulting pooled samples were subjected to size selection and further purification using the Pippin Prep system (Sage Science, Inc., Beverly, MA, United States). The purified libraries were analyzed using the Agilent BioAnalyzer 2100 (Agilent Technologies, Santa Clara, CA, United States) and quantified using the KAPA Library Quant kit (Illumina, San Diego, CA, United States) using real-time PCR. The quantified pooled libraries were sequenced on the Illumina MiSeq platform (Illumina, San Diego, CA, United States) using the V2 500 cycle sequencing kit according to the manufacturer’s instructions using the 250 bp paired end sequencing strategy.

### 2.5 Bioinformatics analysis

The raw paired-end reads were quality-filtered, denoised, and chimeric reads were removed using the DADA2 pipeline (v1.14) (Callahan et al., 2016). Predicted ASVs were assigned to taxonomy using the DADA2-formatted trained SILVA annotation reference database (v138) (Quast et al., 2012). The clearcut function within Mothur (v1.35.1) was used to generate phylogenetic distances between taxa (Schloss et al., 2009). Relative abundances were estimated and log-transformed abundances were generated using the microbiome package (Lahti and Shetty, 2018). Genefilter package, (v1.78.0) (Gentleman et al., 2013) was used to filter ASVs as described previously (Tom et al., 2024) to only retain ASVs present in at least 2 samples with a minimum abundance 0.15% (0.0015 relative abundance) within at least one sample. A read threshold of 6,106 reads was used as the cutoff read depth for the estimation of alpha diversity. The oral and rumen bacterial communities were compared using Observed ASVs and Shannon-Wiener diversity indices of alpha-diversity using the “get_alphaindex” function within the Microbiota Process package (Deng et al., 2023) after rarefying to 6,106 reads. For beta-diversity analysis, Principal Component Analysis (PCoA) was performed using weighted UniFrac and Bray–Curtis dissimilarity matrices using relative abundance information. Non-metric multidimensional scaling (NMDS) was performed using Jaccard distances within the phyloseq package (McMurdie and Holmes, 2013) using relative abundances. The DESeq2 package (Love et al., 2014) was used for differential abundance analysis after log transformation. The Benjamini-Hochberg false discovery rate-adjusted *p-values* were used at < 0.05 to identify significantly different ASVs.

### 2.6 Modeling of host phenotype

Adjusted weaning weight is an economically important trait for beef cattle producers and as such, was chosen as the phenotype of interest in this study. Adjusted weaning weight is the weight of a calf at weaning adjusted for sex, the calf’s age in days (adjusted to 205 days of age), the dam’s age in years, and heterosis of the calf. Only animals with rumen and oral samples and phenotypes were considered for this analysis (*n* = 112). For each sample type, only the ASVs present in at least 5% of animals were considered (225 and 1,353 for oral and rumen data, respectively). Within sample type (oral or rumen), ASVs were normalized by adding one to all reads, calculating the relative abundance of each ASV within a sample, and then applying a log transformation (Yerke et al., 2024). The log-transformed relative abundances were then scaled within a ASV to create a microbial matrix. To estimate variance components, these microbial matrices were used in a Bayesian ridge regression model (Pérez and de los Campos, 2014). The microbial effect was assumed to follow a normal distribution and scaled inverse Chi-squared distributions were assumed for the microbial and residual variances. The BLGR package (Pérez and de los Campos, 2014) in R (v4.2.3) (R Core Team, 2017) was used to fit the model with 12,000 iterations in a Markov chain Monte Carlo algorithm with the first 2,000 discarded as burn-in. All other values were left as defaults.

### 2.7 Statistical analysis

All statistical analysis was performed using R (v4.2.3) and results were visualized using ggplot2 (Wickham and Wickham, 2016). Tukey’s *post hoc* test was used for pairwise comparison within R package Ismean (Lenth and Lenth, 2018). Wilcoxon rank-sum test was used to identify alpha-diversity differences in observed ASVs and Shannon-Wiener index. Permutational Multivariate Analysis of Variance (PERMANOVA) (Adonis function in R package vegan) (Dixon, 2003) was used to identify differences in beta-diversity. Benjamin-Hochberg FDR (false discovery rate) correction for multiple tests was used before identifying differentially abundant ASVs. NMDS analysis of the oral and the rumen bacterial communities were performed using the Jaccard distance, weighted UniFrac distances, and Bray–Curtis dissimilarity matrix to identify between sample variation due to species diversity (Jaccard distance), species diversity and abundance (Bray–Curtis dissimilarity matrix) and species diversity and abundance and phylogenetic relationships (weighted UniFrac distances). Codes and pipelines used in this study are available at the https://github.com/FernandoLab/Bovine-oral-microbiome. Raw sequence reads are available at NCBI’s sequence read archive under accession number PRJNA1159382.

## 3 Results

**Figure 2:**
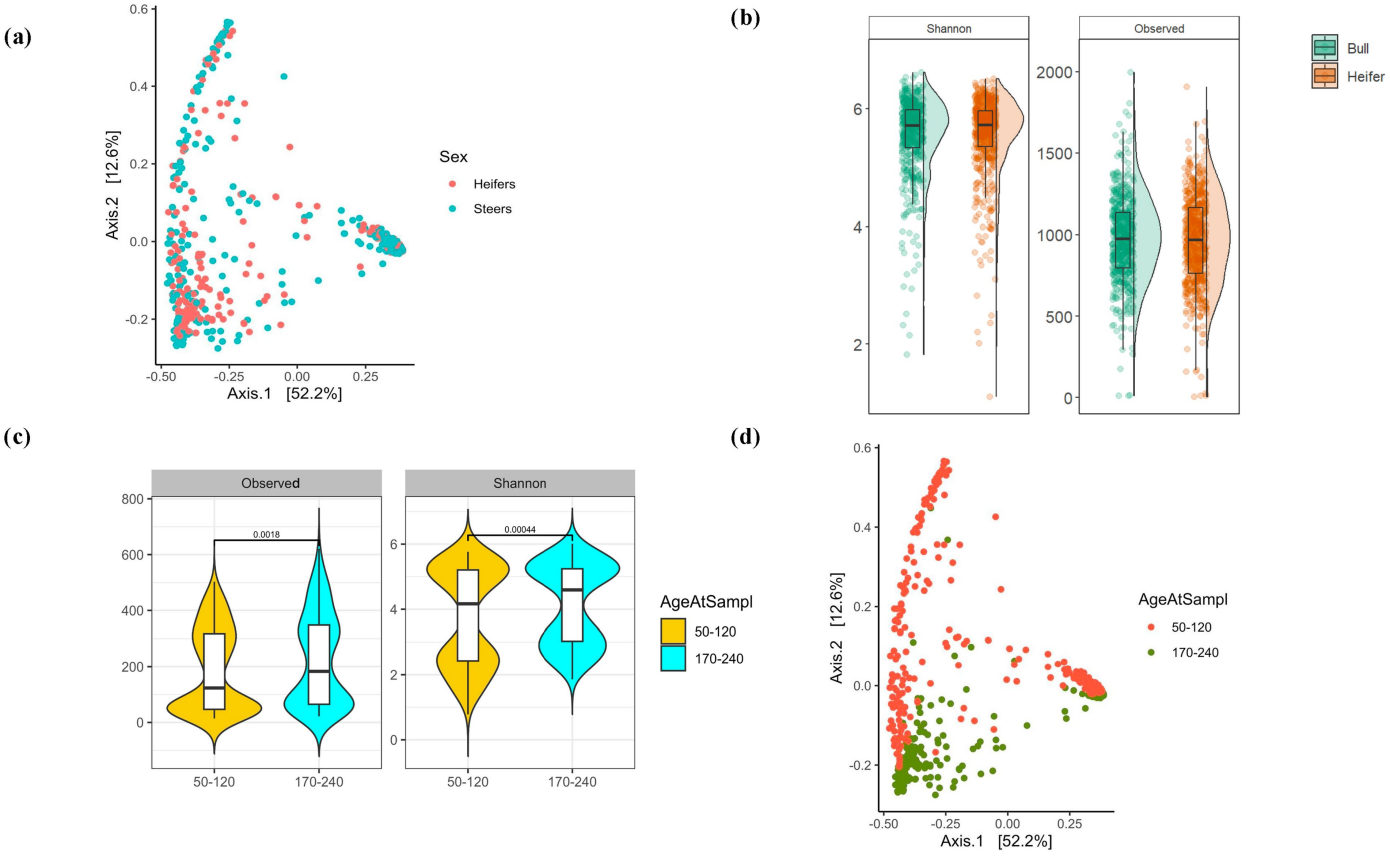
Effect of sex in bacterial community assembly: **(a)** Principal coordinate analysis (PCoA) between heifers and steers/bulls. **(b)** Shannon-Wiener diversity indices and observed ASVs of alpha diversity between heifers and steers. **(c)** Observed ASV and Shannon-Wiener alpha diversity of heifers and steers over time. **(d)** Principal coordinate analysis (PCoA) of the bacterial community composition in heifers and steers over time. Only the first 2 time points was used as the last 2 time points have either heifers or steers.

With paucity of studies investigating the establishment of the rumen and oral bacterial community in beef cattle and the limited information on the applicability of the oral bacterial community as a proxy for rumen bacterial community composition, this study was designed to characterize the assembly of the rumen and oral bacterial communities in beef calves and to evaluate the potential of using the oral bacterial community composition as a proxy for the rumen bacterial community composition. A comprehensive dataset of oral and rumen samples from 166 animals over 4 time points, comprising 71 bulls, 26 Steers (castrated bulls out of the 71 animals) and 95 heifers, across four distinct sampling time points was analyzed to compare bacterial community composition between sampling locations over time (Figure 1a). To this end, we evaluated the establishment and persistence of bacterial species in the rumen and oral bacterial communities in beef cattle using 16S rDNA-based amplicon sequencing. After quality filtering, the dataset contained 3,434 normalized high-quality ASVs from 843 samples, with an average read depth of 17536.43 ± 5,458 reads per sample, accounting for 14,783,209 reads, after filtering using the criterion of minimum abundance of 0.15% within a sample and prevalence in at least two samples (Tom et al., 2024). This included 6,827,910 reads from 422 oral samples and 7,955,299 reads from 421 rumen samples (Figure 1b). Of the 3,434 ASVs identified, 3,395 were assigned to bacteria and 39 to archaea. Phylum-level classification of the bacterial community for both the oral and rumen are shown in Figure 1c. Detailed taxonomic assignment of each ASV is shown in Supplementary Table S1. With the cohort composed of both heifers and bulls, the analysis for sex-associated effects in the bacterial community structure displayed no effect of sex on alpha and beta diversity (Figures 2a–d) (Shannon-Wiener alpha diversity index, Wilcoxon rank-sum test, *p* = 0.38 and PERMANOVA, *p* < 0.001; *R*^2^ = 0.0079).

### 3.1 Establishment of the oral bacterial community and colonization dynamics in beef cattle

**Figure 3:**
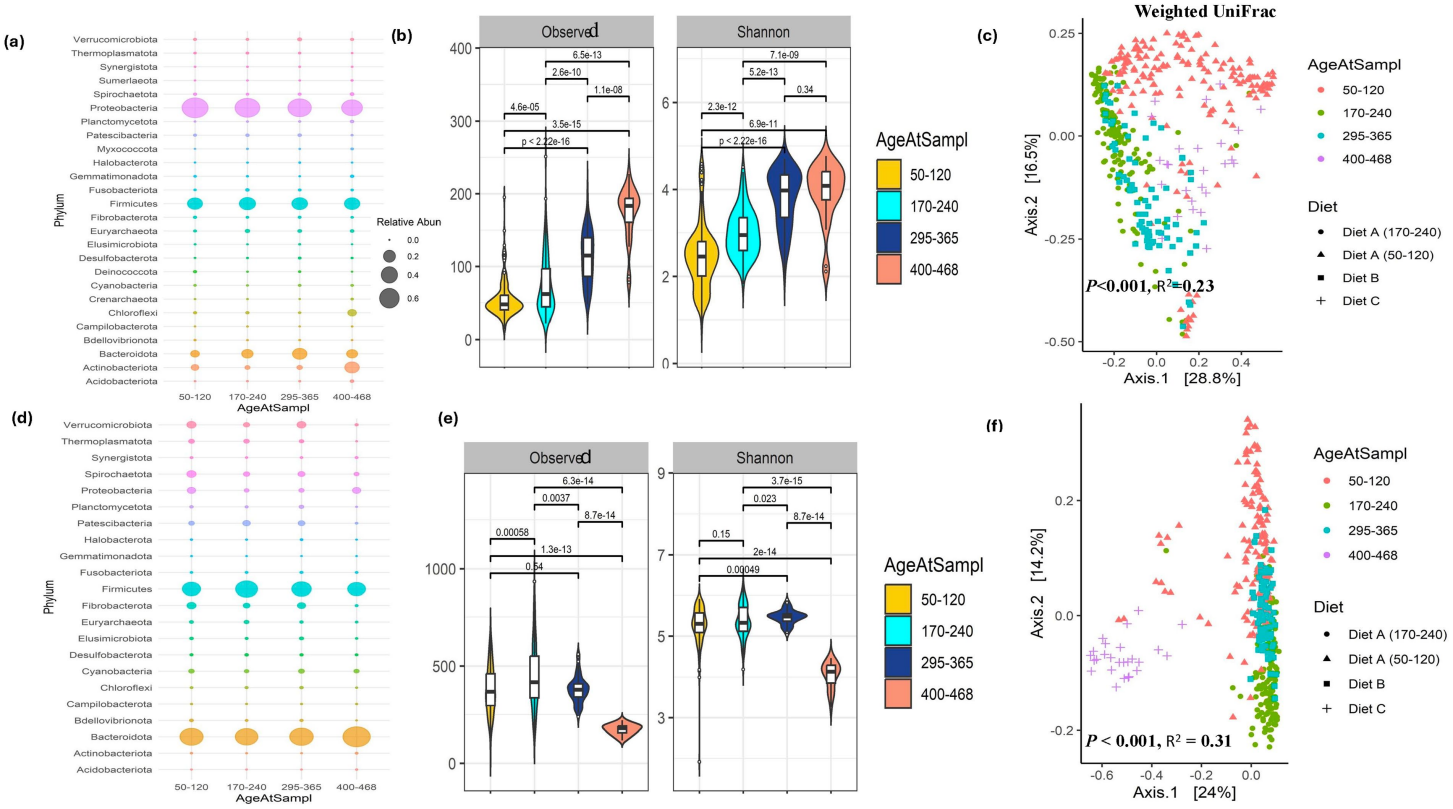
Oral microbiome establishment in beef calves. **(a)** Bubble plot of phylum-level relative abundance distribution in the oral samples with age and dietary change (the first 2 time points, 50–120 days and 170–240 days) the animals are on the same diet and at age of 295–365 days the heifers are on a forage diet and on days 400–468 the steers are on a finishing diet. **(b)** Observed and Shannon-Wiener alpha diversity indices in the oral samples across age. **(c)** Principal coordinate analysis (PCoA) of the oral microbiome at different age and dietary groups. **(d)** Bubble plot of phylum-level relative abundance distribution in the rumen samples with age and dietary change (the first 2 time points, 50–120 days and 170–240 days) the animals are on the same diet and at age of 295–365 days the heifers are on a forage diet and on days 400–468 the steers are on a finishing diet. **(e)** Observed and Shannon-Wiener alpha diversity indices in the rumen samples across age. **(f)** Principal coordinate analysis (PCoA) of the rumen microbiome at different age and dietary groups.

The oral bacterial community was composed of 2,664 ASVs from 422 oral samples, with an average of 16,179 ± 4,989 reads per sample. Of the 2,664 ASVs, 32 were assigned to archaea and 2,632 were assigned to bacteria. At the phylum level, 26 different phyla were identified and are listed in Supplementary Table S2. The mean percentage abundances of each phylum are summarized in Figure 3a. The predominant phyla identified included *Proteobacteria* (61.66%), *Firmicutes* (22.13%), *Bacteroidota* (9.99%), and *Actinobacteriota* (3.46%). The most abundant families in the oral samples were *Pasteurellaceace* (17.52%), *Moraxellaceace* (15.73%)*, Sphingomonadaceace* (14.22%), and *Streptococcaceace* (12.01%). As the animals aged, *Chloroflexi* and *Actinobacteria* increased and were highest at the last sampling. However, since dietary changes occurred post-weaning, it is difficult to know if this change is a diet related change or an age-related change in community. Additionally, *Proteobacteria* decreased with time while Actinobacteriota and Acidobacteriota increase with age. However, as mentioned before this could be a diet related change in the bacterial community than an age-related change. When investigating the development of the oral bacterial community over time, the alpha diversity of the oral bacterial community increased with calf age (Figure 3b). This was also observed in the PERMANOVA analysis which identified significant changes in bacterial diversity across sampling time points that reflect calf age (PERMANOVA, *p <* 0.001, *R*^2^ = 0.23; Figure 3c). However, this increased diversity could be a result of dietary changes that occurred post-weaning. Further analysis of ASVs changing with age using differential abundant analysis identified 70 ASVs to be differentially abundant between the first 2 time points when the animals were on the same diet. As such, these microbes may reflect age-associated bacterial community changes in beef calves. Among the 70 ASVs, we identified 45 ASVs to increase with age while 25 ASVs decreased with age (Supplementary Table S3). We observed an increase in the proportion of fiber digesters with age. The oral samples at the first two time points displayed a significant age-dependent increase in the relative abundance of *Bacteroidota*, *Firmicutes*, *Euryarchaeota*, *Fusobacteriota*, and *Chloroflexi*, while a decrease in *Proteobacteria*, *Actinobacteria*, *Campillobacteria*, *Cyanobacteria*, and *Deinobacteria* was observed. At species-level, many ASVs were identified to be differential over time with *Sphingomonas leidyi*, *Alysiella crassa*, *Mycoplasma bovoculi*, *Rhodococcus erythropolis*, *Streptococcus rupicaprae*, *Ralstonia insidiosa*, *Staphylococcus equorum*, *Aminobacter aminovorans*, *Paenibacillus alginolyticus*, *Lactobacillus amylovorus*, and *Blautia obeum* showing decreased abundance with age (170–240 days of age) as the animal matures and develops a stable oral bacterial community. On the contrary, *Pseudomonas antarctica*, *Stenotrophomonas maltophilia*, *Methylobacterium-Methylorubrum extorquens*, *Christensenellaceae R-7 group*, *Prevotella copri*, *Brevundimonas vesicularis*, *Prevotella ruminicola*, *Mycoplasma hyopneumoniae*, *Bacteroides vulgatus*, *Butyrivibrio fibrisolvens*, *Fusobacterium necrophorum*, *Bifidobacterium adolescentis*, *Streptococcus pluranimalium*, *Achromobacter insolitus*, *Faecalibacterium prausnitzii*, *Phascolarctobacterium faecium*, *Parabacteroides merdae*, *Akkermansia muciniphila*, and *Bacteroides stercoris*, abundance increased with the calf’s age suggesting that such microbes establish as the rumen develops and the calf consumes more forage diets (Supplementary Table S3).

### 3.2 The rumen bacterial community establishment and colonization dynamics

The rumen bacterial community from the cohort consisted of 2,826 ASVs encompassing 7,955,299 reads. These ASVs were classified into 19 bacteria and 3 archaea phyla (Supplementary Table S4). The most represented phyla include *Bacteroidota* (46.95%), *Firmicutes* (33.39%), *Verrucomicrobiota* (3.69%), and *Fibrobacterota* (3.62%) (Figures 1c, 3d). The phyla level changes in the bacterial community displayed an increase in *Bacteroidota* and *Verrucomicrobiota* and a decrease in *Proteobacteria* and *Firmicutes* with age. However, this change could be a result of post weaning dietary changes. *Fibrobacterota* populations increased but were low in the final diet which was a high grain diet. The Observed ASVs and the Shannon-Wiener diversity indices were significantly (Wilcoxon rank-sum test, *p <* 0.001) higher in the first three time points compared to the last time point when the animals were on a high-concentrate diet (Figure 3e). In the principal coordinate analysis, the first three sampling time points clustered away from animals at age 400–468 days, and the PERMANOVA analysis identified a statistically significant difference (PERMANOVA, *p <* 0.001) between the age groups/diet with an *R*^2^ of 31% (Figure 3f). This observation could be a result of dietary changes that occurred post-weaning.

Differential ASV analysis between the first 2 time points of rumen samples (when the animals were on the same diet) identified 112 ASVs to be differentially abundant between the time points. Among the 112 ASVs identified, abundance of 54 ASVs increased with age and the top 5 most differentially abundant taxa included *Unclassified Christensenellaceae R-7 group*, *Unclassified Rikenellaceae RC9 gut group*, *Unclassified Absconditabacteriales (SR1)*, *Butyrivibrio proteoclasticus*, and *Unclassified Pseudobutyrivibrio* (Supplementary Table S5) while 58 ASVs decreased with age such as *Unclassified Bacteroidales RF16 group*, *Fibrobacter succinogenes*, *Sphingomonas leidyi*, *Prevotella copri*, and *Unclassified Fibrobacter* (Supplementary Table S5). Of these, we identified 3 differentially abundant ASVs to be found only in the first time point (50–120 days) belonging to the bacterial species; *Prevotella copri*, *Unclassified Anaerosporobacter*, and *Unclassified Anaerosporobacter* (Supplementary Table S5). In contrast, there were 2 differentially abundant ASVs identified only in the second sampling time point (170–240 days), belonging to species *Stenotrophomonas maltophilia* and *Unclassified Puniceicoccaceae*.

### 3.3 Bacterial community compositional differences between beef cattle oral and rumen

**Figure 4:**
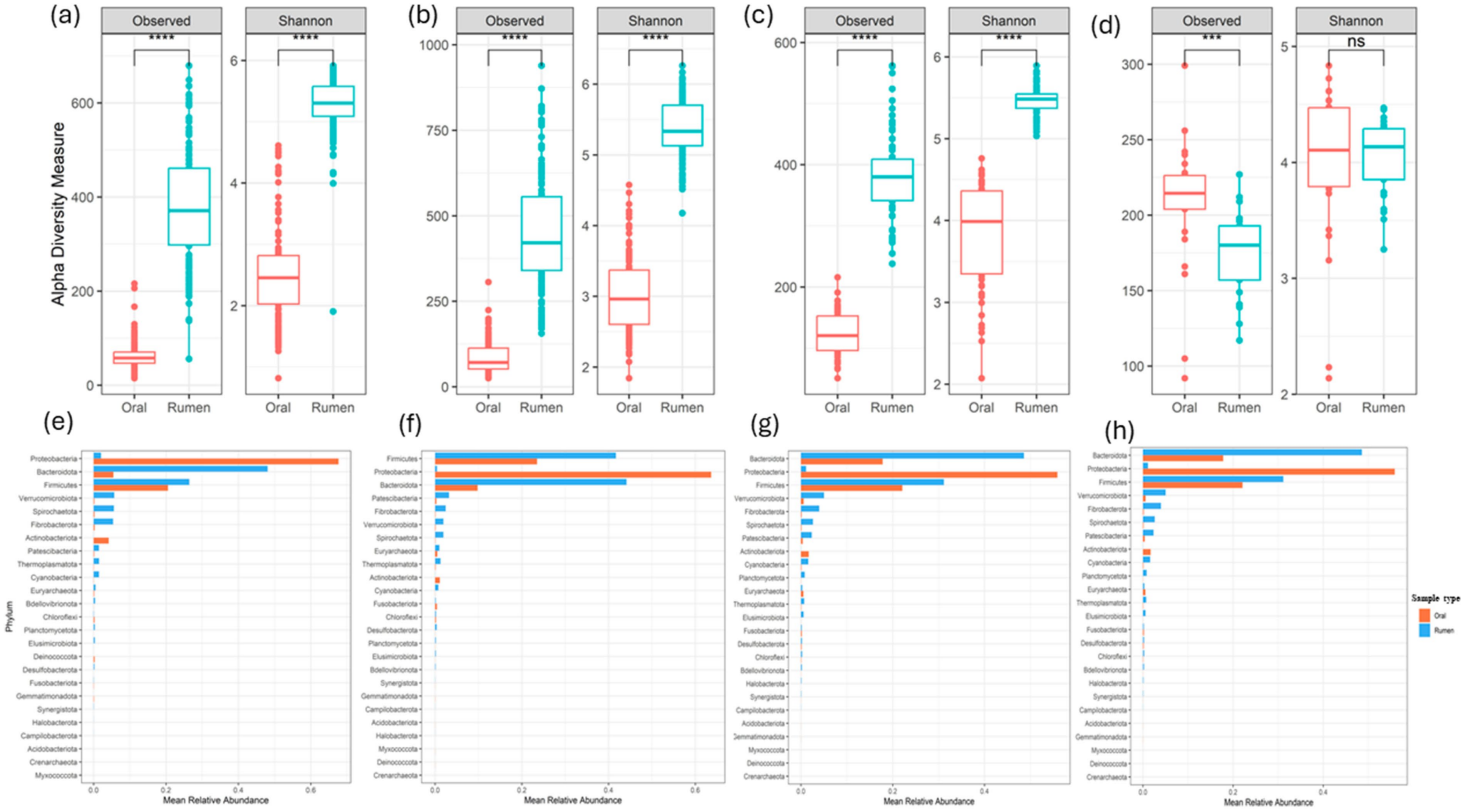
Observed and Shannon-Wiener alpha diversity indices between oral and rumen microbiome at different age groups and diets; 50–120 (milk + forage diet - both heifers and bulls) **(a)**, 170–240 (milk + forage diet - both heifers and bulls) **(b)**, 295–365 (high forage diet - heifers after weaning) **(c)**, 400–468 (high-concentrate diet - steers after weaning) **(d)**. Phylum level-based comparison between oral and rumen microbiome at different age groups and diet; 50–120 (milk + forage diet - both heifers and bulls) **(e)**, 170–240 (milk + forage diet - both heifers and bulls) **(f)**, 295–365 (high forage diet - heifers after weaning) **(g)**, 400–468 (high-concentrate diet - steers after weaning) **(h)**.

**Figure 5:**
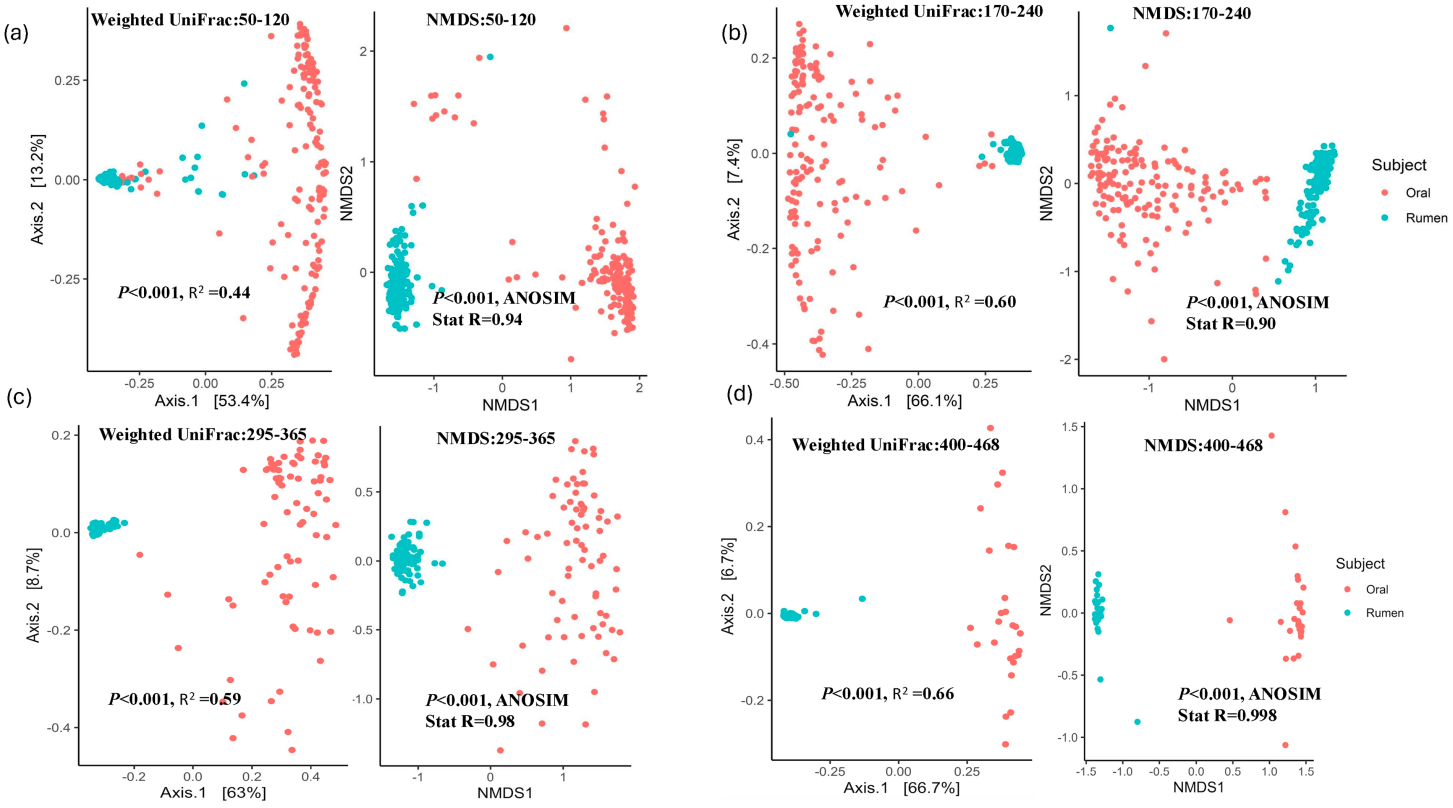
Beta diversity analysis using weighted UniFrac and NMDS (Jaccard distances) between oral and rumen samples at different age groups: 50–120 **(a)**, 170–240 **(b)**, 295–365 **(c)**, 400–468 **(d)**.

**Figure 6:**
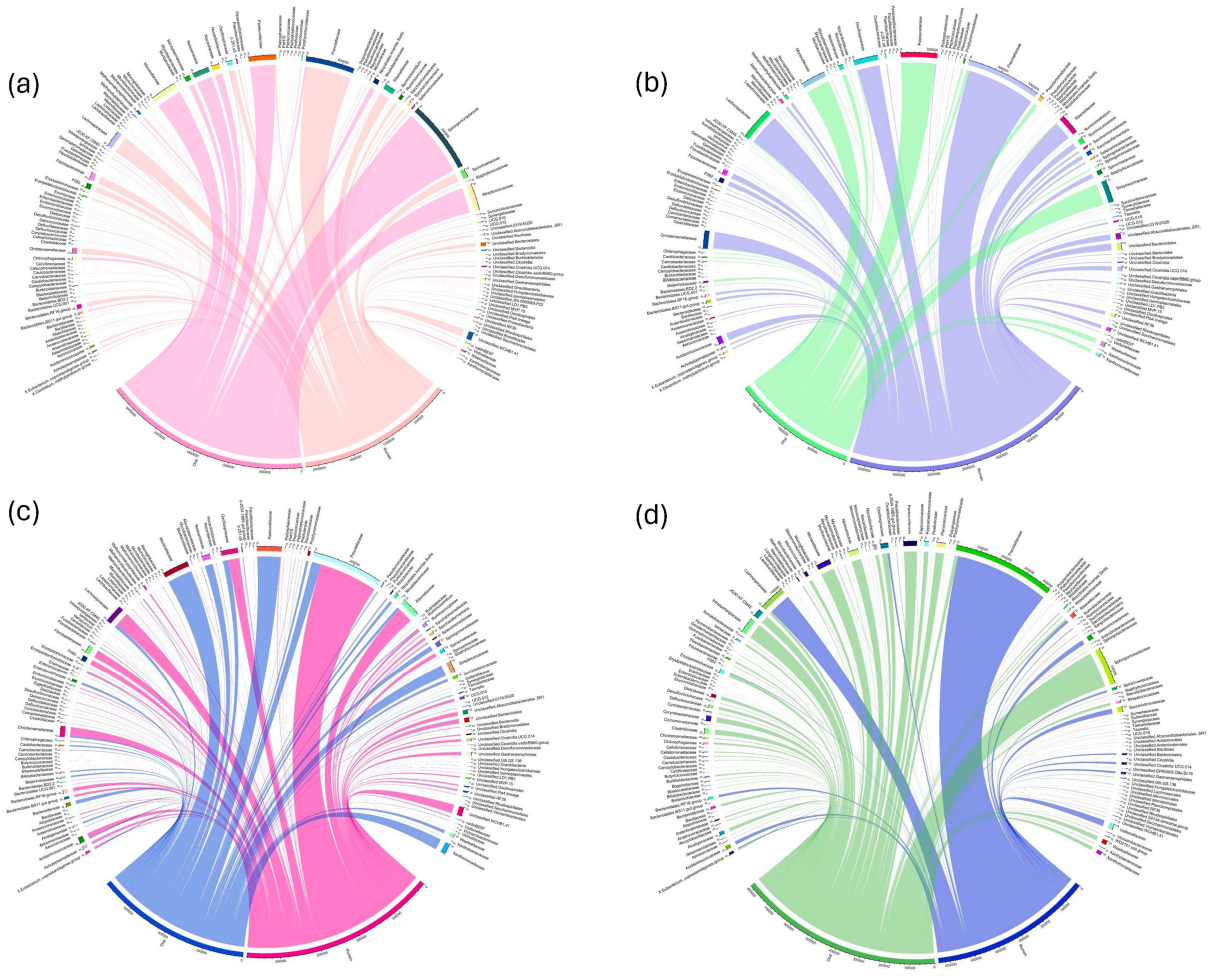
Family level taxonomic abundance distribution between oral and rumen samples at different age groups; 50–120 **(a)**, 170–240 **(b)**, 295–365 **(c)**, 400–468 **(d)**.

As calves matured, there was a notable increase in microbial alpha diversity across age. Since dietary changes occurred during the sampling periods, we limited the comparison between the oral and rumen bacterial community composition to the samples collected at each time point. During the initial three sampling points, when the animals were on a forage diet, the rumen bacterial community exhibited significantly higher diversity (Wilcoxon rank-sum test, *p* < 0.0001) and species richness than the oral bacterial community. Alpha diversity analysis revealed statistically significant differences (Wilcoxon rank-sum test, *p* < 0.0001) in the bacterial community composition between the oral and the rumen samples collected at the first 3 time points investigated (Figures 4a–c). However, this distinction was not apparent in the last time point (400–468 days) (Shannon-Wiener index, Wilcoxon rank-sum test, *p* = 0.67) (Figure 4d). Nonetheless, the oral bacterial community composition remained statistically different from the rumen bacterial community composition based on Observed ASVs (Wilcoxon rank-sum test, *p <* 0.001), as illustrated in Figure 4d. Phyla *Proteobacteria* and *Actinobacteriota* were significantly higher (*t*-test, *p* < 0.0001) in the oral samples compared to the rumen samples. Conversely, significant increases (*t*-test, *p* < 0.0001) in the abundance of *Bacteroidota*, *Firmicutes*, *Verrucomicrobiota*, *Spirochaetota*, *Patescibacteria*, *Thermoplasmatota*, *Cyanobacteria*, and *Fibrobacterota*, were observed in the rumen samples compared to the oral samples (Figures 4e–h). To assess between sample bacterial community differences across timepoints, we utilized weighted UniFrac distance matrix, Bray-Curti’s dissimilarity matrix, and NMDS (Figure 5; Table 1). The weighted UniFrac analysis revealed distinct clustering with significant differences between the oral and rumen samples at all age groups (Table 1). The NMDS analysis using the Jaccard distances was consistent with these findings, showing significant statistical differences between the oral and rumen bacterial communities at all sampling time points (Table 1) as illustrated in Figure 5. To further identify taxonomic differences in the rumen and oral bacterial communities that lead to community differences, we analyzed compositional differences at different taxonomic levels. The most abundant bacterial families identified in rumen and oral bacteriomes are shown in Figures 6a–d.

**Table 1:** Beta diversity analysis of Bray-Curtis, weighted UniFrac, and NMDS between oral and rumen samples at the different age groups.

Time points (days)	Samples	Bray Curtis		Weighted Unifrac		NMDS	
		*R* ^2^	*p*-value	*R* ^2^	*p*-value	ANOSIM Stat R	*p*-value
50-12030-120	Rumen vs. oral	0.26	0.001	0.44	0.001	0.94	0.001
170-2170-240	Rumen vs. oral	0.32	0.001	0.60	0.001	0.90	0.001
295-3295-365	Rumen vs. oral	0.38	0.001	0.59	0.001	0.98	0.001
400-4400-468	Rumen vs. oral	0.44	0.001	0.66	0.001	0.998	0.001

**Figure 7:**
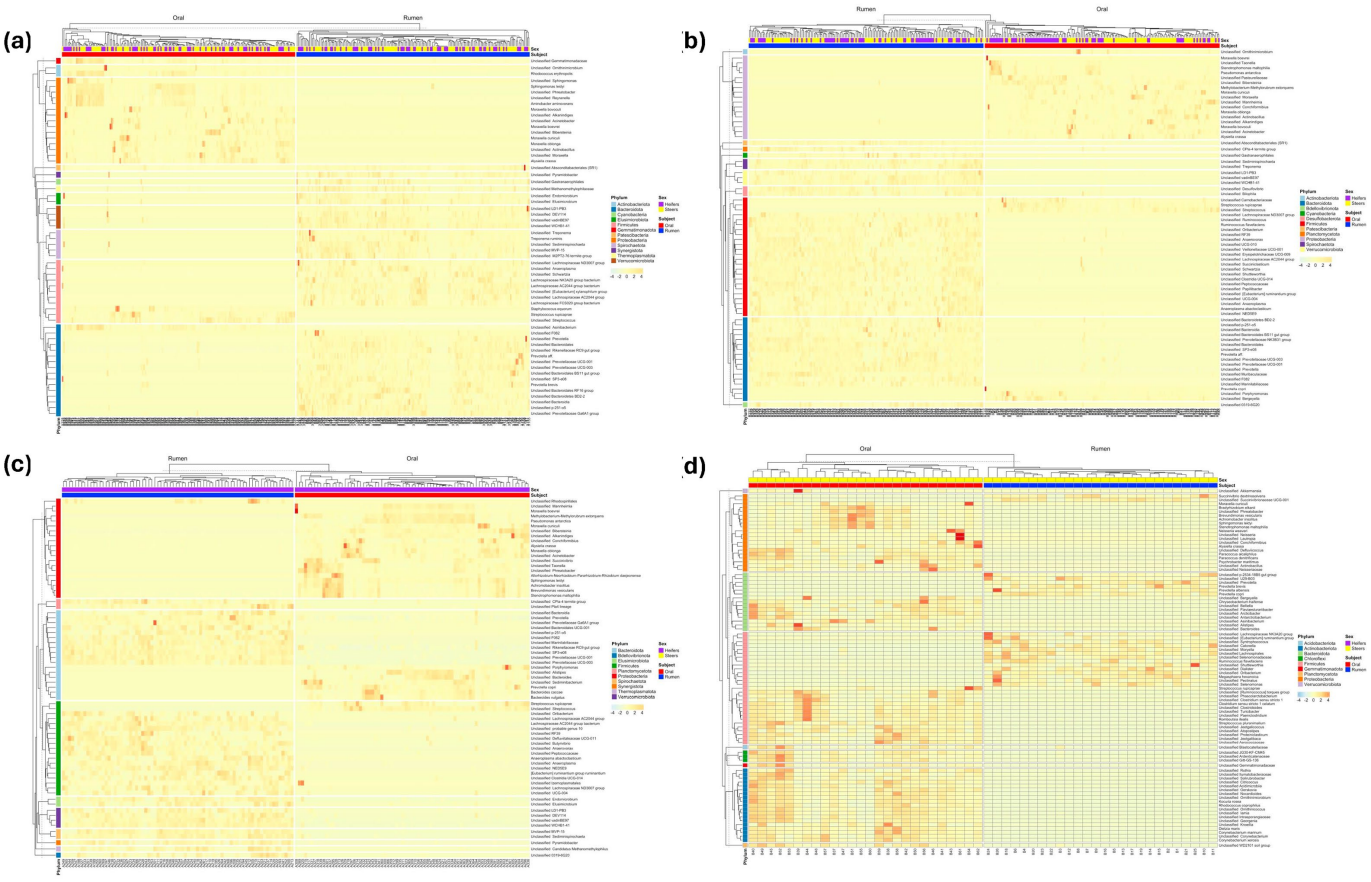
Heatmap of the differential abundance analysis at ASV level. The top 80 most differential abundant ASVs between the oral and rumen samples at the genus level across different age groups; 50–120 **(a)**, 170–240 **(b)**, 295–365 **(c)**, 400–468 **(d)**.

ASV level analysis of differential ASVs identified a large number of ASVs to be differentially abundant between the oral and rumen samples at different sampling timepoints (Figures 7a–d). For 50–120 days of age, 233 ASVs were identified as differentially abundant (Negative binomial model, Benjamini-Hochberg false discovery rate-adjusted, *padj*< 0.05) between oral and rumen samples (Figure 7a; Supplementary Table S6). At 170–240-days of age, 229 ASVs were identified to be differentially abundant between the two sample types (Figure 7b; Supplementary Table S7). At 295–365 days of age, 216 ASVs were identified to be differentially abundant between oral and rumen samples (Figure 7c; Supplementary Table S8). Finally, at 400–468 days of age, when the animals were on a high grain diet, 228 ASVs were identified to be differentially abundant between oral and rumen microbial communities (Figure 7d; Supplementary Table S9).

### 3.4 Capacity to utilize bacterial community composition to explain variation in weaning weight

Although distinctly different bacterial species compositions were observed in both the oral and rumen bacteriomes, we explored the possibility of using the earliest bacterial community composition collected from each sample type to model an early in life growth trait. Utilizing a Bayesian Ridge Regression model, we estimated the proportion of variation explained in adjusted weaning weight of calves using bacterial composition data collected from each location at 50–120 days of age. The proportion of variation explained in host phenotypes by the oral and rumen bacterial community were 30 and 37%, respectively. The credible intervals for the estimates for the oral and rumen bacterial communities were [10, 42] and [15, 50], respectively.

## 4 Discussion

To better understand the role of the rumen microbiome to improve animal health and productivity, animal studies with larger sample sizes are greatly needed. For such studies to take place, noninvasive sampling methods that are more accessible and ethical are greatly needed. Recently, studies have suggested the use of oral swab sampling close to rumination for enumeration of oral bacterial communities as a proxy for the rumen bacterial community (Kittelmann et al., 2015; Edwards et al., 2023). Given that ruminant animals frequently pass a substantial portion of rumen content into the oral cavity for mastication, physiologically, utilizing the oral bacterial community as proxy for rumen bacterial community could be justified. However, small pilot studies comparing the oral bacterial community to rumen bacterial communities have resulted in varied conclusions (Kittelmann et al., 2015; Tapio et al., 2016; Young et al., 2020; Miura et al., 2022; Amin et al., 2023). Kittelmann et al. reported that differences in bacterial community clustering were primarily due to dietary variations rather than sampling methods, after accounting for oral bacterial community composition in oral swabs of sheep (Kittelmann et al., 2015). Additionally, most studies comparing the oral and rumen bacterial communities have utilized higher taxonomic level comparisons such as phylum or family level (Amin et al., 2021; Mott et al., 2022), where different species can belong to the same phyla. As such, to evaluate if the oral bacterial community composition can be used as a proxy for the rumen, bacterial community analysis needs to be performed at the lowest taxonomic level at high resolution. To this end, in this study, we compared the oral and rumen bacterial communities from beef calves over 4 time points under different diets at the ASV level to evaluate the use of the oral bacterial community as a proxy for the rumen bacterial community composition. The 4 time points were evaluated to identify if there is a window where the oral bacterial community can be used as a proxy. Additionally, here we describe the colonization of the oral and rumen bacterial communities in beef calves and finally evaluate the applicability of using the oral bacterial community composition to explain variation in weaning weight.

### 4.1 The bovine oral bacterial community establishment and dynamics

The oral cavity harbors a dense microbial community (Grassl et al., 2016). The relationship between the oral microbiome and human health is well documented, with evidence suggesting a predictive relationship between the oral microbiome and various human diseases, making the oral cavity an ideal site for disease diagnosis (Xiao et al., 2020). Additionally, the oral cavity’s accessibility allows for non-invasive collection of biological samples (Boutaga et al., 2007). The alpha diversity analyses revealed that oral bacterial diversity increases with age and that dietary modulations further contribute to the diversity and species richness of the oral bacterial community. During the first two time points, the animals were maintained on the same diet, thus allowing the opportunity to identify bacterial taxa that change with age. Differential ASV analysis between these two-time points revealed that 126 ASVs to increase with age. At the species level, the oral bacterial community of calves was initially dominated by bacteria such as *Streptococcus* spp., *Prevotella* spp.*, Blautia obeum*, *Bacteroides heparinolyticus*, *Butyrivibrio proteoclasticus*, *Fusobacterium nucleatum*, *Anaerostipes* spp., *Phascolarctobacterium faecium*, *Akkermansia muciniphila*, *Bacteroides* spp., *Bifidobacterium adolescentis*, *Actinobacillus pleuropneumoniae*, *Methylobacterium-Methylorubrum extorquens*, *Faecalibacterium prausnitzii*, and *Brevundimonas vesicularis* capable of degrading lactose, starch and other polysaccharides, which were later replaced or dominated by fiber-degrading bacteria. Amin et al. reported that the fecal bacterial community in young calves was initially dominated by lactose and starch degraders and was eventually replaced by fiber degraders (Amin et al., 2023). Similar studies have observed age-related decreases in the abundances of *Bifidobacterium*, *Lactobacillus*, and *Faecalibacterium* (Dill-McFarland et al., 2017; Virgínio Júnior et al., 2021) and age-related increase in fiber-degrading *Ruminococcus* (Virgínio Júnior et al., 2021). We observed an age-dependent increase in potential pathogenic bacteria such as *Streptococcus dysgalactiae*, *Fusobacterium necrophorum*, *Fusobacterium nucleatum, Streptococcus dysgalactiae*, *Streptococcus pluranimalium*, *Moraxella boevrei*, *Moraxella cuniculi*, and *Moraxella macacae*, suggesting that the oral microbiome may be a reservoir for opportunistic pathogens in ruminants. As such, studying the oral microbiome may provide information on the animal’s health status. Additionally, this change in the oral bacterial community composition may be a consequence of the young calf transitioning from milk to a forage diet and physiological changes associated with rumen development and the onset of rumination.

The beta diversity analyses of the oral bacterial community identified no distinct clustering between different ages or dietary groups, however, the PERMANOVA analysis identified significant differences (PERMANOVA, *p <* 0.001) in bacterial community structure by sampling time point. As the calf grows, the substrates the animal is exposed to also changes, as a result, microbes can increase based on substrate availability and also due to environmental exposure to microbes such as microbes in the soil during grazing through soil ingestion. As such, the oral microbiome in the young calf may be highly dynamic and may be susceptible to rapid changes in the animal and the environment. Similar to our observations, previous studies have reported that most commonly found bacterial phylum in the gut and oral cavity are members of the phylum *Proteobacteria* (Kado et al., 2020). However, the observed decrease in the proportion of *Proteobacteria* with age in this study and others may be due to the calves transitioning from milk to grazing and the onset of rumination. As such, the increased exposure to plant fibers may have increased fiber digesters and decreased *Proteobacteria* abundance. An increase in the phylum *Actinobacteriota* was observed at the final time point when the animals were on a high-starch diet. Previous studies have reported subtle diet-related differences in the oral microbiota between treatment groups (Kittelmann et al., 2015). As such, the increase in *Actinobacteria* may be a result of dietary change. We identified 5 oral-associated bacterial ASVs to be persistent in all the oral samples across sampling time points. This included, *Unclassified Bibersteinia* (ASV_3), *Unclassified Streptococcus* (ASV_4), *Alysiella crassa* (ASV_5), *Unclassified Actinobacillus* (ASV_58), and *Moraxella oblonga* (ASV_7). These persistent ASVs accounted for higher proportions of the total reads at each time point suggesting that they are important oral-associated bacteria. Consistent with our findings, Kittelmann et al. reported that the bovine oral microbiota was dominated by typical oral bacteria, including *Actinobacillus*, *Bibersteinia*, and *Moraxella*, among others (Kittelmann et al., 2015). We believe that the oral bacterial community establishment is affected by age, substrates utilized by the animal as well as early microbes that colonize the oral cavity. Our data indicates that the early oral bacteriome in bovines is initially dominated by the phylum *Proteobacteria* when the animals are on a milk diet. As the animal transitions to a forage-based solid diet, the substrates available to the oral microbes change. As a result, the substrate availability changes leading to colonization of fiber digesters and other microbes, including potential pathogenic species. This process reflects the colonization wave model proposed for gut microbial colonization (Gharechahi et al., 2020). However, factors including host genetics, animal health, substrates available, and colonization history may all influence oral bacterial community establishment (Fernando et al., 2025). We believe oral bacterial community colonization is driven by both the introduction of new species and the proliferation of early colonizers. Moreover, the oral bacteriome demonstrates considerable dynamism, responding rapidly to dietary and physiological changes. This study is the first to characterize the colonization dynamics of the bovine oral bacterial community over time. However, since dietary changes occurred post-weaning, it is difficult to know if some of the changes that occurred after weaning are diet related changes or age-related change in community.

### 4.2 The bovine rumen bacterial community establishment and dynamics

The modulation of early rumen microbiota has emerged as a strategy to enhance the benefits of ruminant agriculture and mitigate its environmental impacts (O’Hara et al., 2020). Therefore, understanding the dynamics of rumen microbial colonization is crucial to identify the optimal time for microbiome intervention to improve animal health and productivity. Previous studies have demonstrated that ruminant animals harbor a core group of successional species that colonize early and persist into adulthood (Furman et al., 2020). In this study, the bacterial community composition changed as the animal aged, and the diet changed. This change in both the alpha and beta diversity of the ruminal bacterial community is a combination of age effects and dietary effects allowing the opportunity to identify age related and diet related bacterial community changes. The marked increase (Wilcoxon rank-sum test, *p <* 0.001) in Observed ASVs and Shannon-Wiener diversity indices in the ruminal content of animals during the first three sampling time points show how age-related changes in the rumen bacterial community occurs on a forage diet. Previous studies have shown that ruminal fermentation varies with age and is linked to age-related shifts in rumen microbial taxa (Yin et al., 2021). Similar to previous reports (Jami et al., 2013; Jami et al., 2014; Newbold and Ramos-Morales, 2020), we identified age-associated increase in rumen bacterial taxa abundance. Yin and colleagues reported that changes in microbial genera associated with age were correlated with volatile fatty acid concentrations and microbial crude protein levels in the rumen (Yin et al., 2021). Specifically, they identified positive correlations between volatile fatty acids and *Prevotella* spp., *Lachnospiraceae NK3A20 group*, *Ruminococcus gauvreauii*, *Ruminococcaceae UCG-014*, and *Ruminococcus* spp. (Yin et al., 2021) many of which were identified in this study.

The increase in alpha diversity could be a result of increased substrate diversity as the animals transition from milk to a forage diet. Additionally, the diet quality may have changed over time and may have contributed to the microbial changes observed. Many studies have demonstrated that high-forage diets have increased bacterial diversity (Fernando et al., 2010; De Menezes et al., 2011; Anderson and Fernando, 2021). However, when the steers were fed a high-concentrate diet (days 400–468), the bacterial diversity significantly decreased. This may be a result of the highly fermentable substrates in the high starch diet that leads to overdominance of starch utilizers resulting in decreased pH that reduces growth of fiber digesters and many other microbes leading to decreased diversity (Fernando et al., 2010; Petri et al., 2012; Grilli et al., 2016; Zhang et al., 2018; Liu et al., 2019). The ruminal microbiota composition at the first three time points was similar and distinctly clustered away from that of animals at 400–468 days of age. We believe this is a result of the high-concentrate diet fed to the animals at the later stage and demonstrates diet related changes in the rumen bacterial community. The rumen bacterial community of calves was predominantly composed of phyla *Bacteroidota*, *Firmicutes*, *Verrucomicrobiota*, *Spirochaetota*, and *Fibrobacterota*. Similar to our observations, studies have reported that the *Bacteroidetes* phylum was more dominant during the first few months after birth followed by an increase in *Firmicutes* (Kittelmann et al., 2015; Furman et al., 2020). It has been proposed that the establishment of the rumen microbiome occurs in successive waves (Gharechahi et al., 2020) and our data supports this notion. Here we demonstrate that both age and diet play a role in rumen bacterial community establishment where the initial colonization in the calf is dominated by *Proteobacteria* (70%) and *Bacteroidetes* (14%), and *Pasteurellaceae* as the predominant family (58%). These early rumen colonizers may have entered during birth or soon after from the environment and are likely important to the newborn calves health and development (Yin et al., 2021). As the animal transitions to forage-based diets and develops a functional rumen, *Bacteroidota*, *Firmicutes*, *Verrucomicrobiota*, *Spirochaetota*, and *Fibrobacterota* populations increase. Similar to our findings, Yin et al. reported age-related increases in the relative abundances of *Firmicutes* and *Bacteroidota* and an age-related decrease in the relative abundances of *Proteobacteria* (Yin et al., 2021).

### 4.3 Is the oral bacterial composition a good proxy for the rumen bacterial composition?

Previous studies have suggested that the bovine oral bacterial composition could potentially serve as a proxy for the bovine rumen bacterial composition (Kittelmann et al., 2015; Tapio et al., 2016; Indugu et al., 2021). Here, using ASV level comparisons, we investigated oral and rumen bacterial community composition in a cohort of beef calves over multiple time points and diets. The Observed ASV and Shannon-Wiener diversity indices indicated that the rumen bacterial community was significantly different (Wilcoxon rank-sum test, *p* < 0.0001) between the oral and rumen bacterial communities at the first three sampling time points. This observation of alpha diversity differences between the oral and rumen bacterial community composition has also been reported previously (Young et al., 2020; Amin et al., 2021). At the last sampling, when animals were on a high-concentrate diet, the Observed ASV alpha diversity index was significantly different (Wilcoxon rank-sum test, *p <* 0.001), while the Shannon-Wiener index was not significantly different (Wilcoxon rank-sum test, *p* = 0.67) between the oral and rumen bacterial communities. This may be due to the lower animal numbers on the finishing diet compared to the 3 earlier time points. Beta diversity indices, including weighted UniFrac, NMDS, and Bray-Curtis, all suggested that the oral bacterial community was significantly different (PERMANOVA, *p <* 0.001) from the rumen bacterial community. Previous studies have also demonstrated significant differences between oral swab samples and esophageal tube samples (Kittelmann et al., 2015; Young et al., 2020; Amin et al., 2021). These global differences between the oral and rumen bacterial communities may be due to microenvironments within each location, the aerobic nature of the oral microbiome compared to the anaerobic rumen microbiome, substrate diversity, and host physiological factors.

To identify bacterial community members that are different between the oral and rumen bacterial communities, we performed differential analyses at different taxonomic levels. The oral and rumen bacterial communities were both taxonomically and compositionally distinct. The phyla *Proteobacteria* and *Actinobacteriota* were significantly more abundant (*t*-test, *p <* 0.0001) in the oral bacterial community compared to the rumen. This increase could be attributed to the animals being on a milk diet, as these phyla include various families and genera of microbes that facilitate the efficient utilization of milk components, supporting a healthy gut transition. In contrast, *Bacteroidota*, *Firmicutes*, *Verrucomicrobiota*, *Spirochaetota*, and *Fibrobacteriota*, were significantly more prevalent in the rumen samples compared to the oral samples (*t*-test, *p* < 0.0001). The abundance of these rumen microbes increases in response to the complex carbohydrates in the calf’s diet, particularly forage associated nutrients like cellulose, hemicellulose, and pectin. Previous studies have reported that the oral microbiota has a higher prevalence of *Pasteurellales* (65.6%) and *Lactobacillales* (26.6%), representing approximately 90% of the total reads (Mott et al., 2022). Similarly, we detected higher abundance of *Pasteurellaceae*, *Moraxellaceae*, *Sphingomonadaceae*, *Streptococcaceae*, and *Neisseriaceae* in the oral bacterial community. In contrast, the rumen bacterial communities were dominated by rumen-associated bacterial families such as *Prevotellaceae*, *Lachnospiraceae*, *Rikenellaceae*, *Oscillospiraceae*, and *Christensenellaceae*. Previous studies have reported the recovery of rumen-associated microbes in oral swab samples after filtering out oral-associated bacterial communities (Kittelmann et al., 2015). Similarly, we identified rumen-associated bacterial families such as *Prevotellaceae*, *Oscillospiraceae*, *Christensenellaceae*, *Rikenellaceae*, and *Methanobacteriaceae* in the oral samples; however, they were identified at relatively low proportions compared to oral-associated microbes and did not represent the compositional abundance found in the rumen. The presence of these typical rumen-associated microbial communities in the oral samples could be due to the onset of ruminations and regurgitation. As such, although some rumen bacteria can be recovered from the oral bacterial community, the abundance of such bacteria and archaea are different from what is observed in the rumen. Therefore, in this study using oral swabs as a proxy for rumen bacterial composition does not appear to be representative of the true bacterial community composition and diversity within the rumen. Young et al. investigated the best sampling time to use oral samples as a proxy for rumen sampling. Although with low animal numbers they also observed oral samples to cluster away from rumen samples with closer clustering in the sampling just prior to feeding (Young et al., 2020). Based on this observation, they suggested best sampling is prior to morning feeding. Therefore, we collected the oral and rumen samples from feedlot steers just prior to feeding, however, the bacterial communities were significantly different between the oral and rumen samples suggesting even just before feeding the oral samples are not a good proxy for the rumen sampling. The reason that the study by Young et al. did not see differences may be due to small sample size of 8 animals. Previous studies reported that oral swabs and bolus samples could not replace rumen sample collection due to the significant differences in both taxonomy and composition (Kittelmann et al., 2015; Tapio et al., 2016; Young et al., 2020; Amin et al., 2021; Miura et al., 2022). The oral cavity is the first body site to encounter ingested feed and exogenous microbes before they enter the gastrointestinal and/or respiratory tracts (Dutzan et al., 2016). While most studies have focused on identifying the presence and proportion of rumen-associated microbiota by comparing taxa at phylum and family level in oral and rumen samples, species or sub-species level analysis has not been performed. Therefore, we performed compositional and differential analysis at ASV level to identify bacterial community differences between the oral and rumen samples. Our analysis revealed that at each time point, the bovine oral bacterial community was dominated by typical oral-associated microbial communities such as *Sphingomonadaceae*, *Moraxellaceae*, *Streptococcaceae*, *Pasteurellaceae*, *Xanthomonadaceae*, *Instrasporangiaceae*, and *Neisseriaceae* while the rumen bacterial community was dominated by typical rumen-associated communities such as *Prevotellaceae*, *Lachnospiraceae*, *Rikenellaceae*, *Fibrobacteraceae*, *Spirochaetaceae*, *Oscillospiraceae*, *Christensellaceae*, *Veillonellaceae*, *Succinivibrionaceae*, and *Selemononadaceae*, as the animals aged. This highlights distinct trajectories of bacterial community colonization at each site. At each time point, the oral bacterial community exhibited a greater number of differential ASVs with higher abundance compared to the rumen bacterial community, possibly reflecting an increase in microbial diversity in the oral bacteriome with age. These differential ASVs in the oral samples represent species capable of fermenting various mono and disaccharides such as glucose, lactose, cellobiose, sucrose, maltose, fructose, arabinose, and maternal milk oligosaccharides (LoCascio et al., 2007; Marcobal et al., 2010; Yu et al., 2013). At the genus level, taxa such as *Lactobacillus*, *Sediminispirochaeta*, *Bifidobacterium*, *Proteiniclasticum*, and *Deinococcus* were prominent. In contrast, differential ASVs associated with the genera *Treponema*, *Fibrobacter*, *Anaeroplasma*, *Eubacterium ruminantium group*, *Ruminococcus*, unclassified *Hungateiclostridiaceae*, *Anaerosporobacter*, *Methanobrevibacter*, and unclassified *Methanomethylophilaceae* were exclusively found in rumen samples. The proportion of these rumen-associated ASVs increased as the animal aged and were influenced by dietary supplementation, particularly as they transitioned from a predominantly milk based diet to low- and high quality forage diets and to high-concentrate diet. These dietary changes significantly alter the substrates available to the rumen microbiota, potentially affecting microbial community composition, function, and fermentation end-products (Petri et al., 2013). Our data clearly demonstrates that the oral and rumen bacterial communities are compositionally distinct and oral swab samples cannot be used as a proxy for rumen bacterial community structure and composition.

A study published by Tapio et al. reported the oral bacteriome to be a good proxy to estimate rumen bacterial diversity (Tapio et al., 2016) which is contradictory to this study. However, in the study by Tapio et al., rumen samples collected were filtered through 2 layers of cheesecloth. This may have limited the number of particle associated microbes in the rumen. Studies have suggested that approximately 70–80% of microbial organic matter is associated with the particulate phase of the rumen contents (Craig et al., 1987), and the filtration using the cheesecloth may have limited the number of particle associated microbes in the sample. Therefore, the comparison may have been made between rumen fluid associated bacterial community and the oral bacterial community rather than total bacterial community in the rumen as utilized in this study. Additionally, similar to this study, as study by de Freitas et al. compared esophagealy tubed whole rumen content samples to saliva samples and reported that rumen bacterial community is distinctly different from the oral bacterial community. Therefore, it is critical to ensure proper sampling that represent each microbial community for comparison.

All current studies investigating the oral and rumen bacterial communities have compared bacterial communities from animals in a confined space where intake can be controlled and samples collected relative to feeding. However, no study has attempted to investigate if oral bacterial community samples would represent rumen bacterial community in grazing settings. To our knowledge this is the first study investigating oral and rumen bacterial communities in a grazing setting and we show that in addition to confinement settings, in grazing settings where rumination is highest, the oral bacterial composition is significantly different from the rumen bacterial composition. Finally, as with many 16S rRNA sequencing studies, short read sequencing limits classification of bacterial taxa to species level due to the short read length. Therefore, in this study we performed all analysis at ASV level and reported the lowest taxonomic level for each ASV that can be classified with high confidance.

### 4.4 Applicability of using bacterial composition to explain variation in weaning weight

Rumen microbial communities and their genes have been leveraged to predict various host associated production traits (Ross et al., 2013; Gebreyesus et al., 2020), including associations with feed efficiency (Shabat et al., 2016; Li and Guan, 2017), methane production (Wallace et al., 2015; Difford et al., 2018), milk yield and milk protein levels (Jami et al., 2014), and overall health (McCann et al., 2016). However, the invasiveness and logistical challenges of rumen sample collection (Huws et al., 2018; Fortina et al., 2022; Hagey et al., 2022) have constrained the number of animals that can be included in these studies, thereby limiting the predictive power of rumen microbiota on host phenotypes. Although we clearly demonstrate that the oral bacterial community composition is not a good proxy for rumen bacterial community structure, here we evaluated if the oral bacteriome is associated with weaning weight and if we could utilize the oral bacteriome to explain variation in weaning weight, and ultimately predict, economically important production traits such as weaning weights. Researchers have suggested that the structure and composition of the oral microbiome could be used to predict the establishment of rumen microbiota communities (Kittelmann et al., 2015). A recent study demonstrated that the oral microbiome might be more effective in predicting feed intake in dairy cows than the rumen microbiome (Marcos et al., 2024). Most of these studies have compared oral and rumen microbiota while accounting for typical oral-associated microbes (Kittelmann et al., 2015; Amin et al., 2021). Therefore, we explored the potential of early oral microbiome sampling to explain variation in adjusted weaning weight. Our findings suggest that the oral bacteriome has the potential to predict host traits, although at a lower accuracy than the complementary rumen bacterial community composition. This may be because traits like weaning weight are more directly influenced by the rumen microbiota and the metabolic products of rumen fermentation. Given that the most typical oral microbiota often includes potential pathogens, and that the oral cavity is continuously exposed to exogenous microbes, the oral microbiome may offer better predictability for host health. While the rumen microbiome appears to be a stronger predictor for production traits, there is value in exploring the oral microbiome’s potential for predicting other phenotypes, such as feed intake and animal health. The sample size for predicting weaning weight in this study was relatively small and additional work in future studies should focus on evaluating the oral microbiome’s utility in predicting animal health and intake in larger populations. Additionally, the associations observed need to be experimentally validated to ensure causation.

## 5 Conclusion

This study investigated the colonization of the oral and rumen bacterial communities, comparing the establishment of bacterial communities in oral swabs and rumen content samples obtained via esophageal tubing from a cattle population at four developmental stages across different diets. Our findings indicate that both rumen and oral colonization occur early, with bacterial diversity increasing with age. Our results also suggest that the oral and rumen bacterial communities are distinct, and the oral bacterial community composition may not serve as a proxy for the rumen bacterial community structure and composition at any stage of the animal’s life. However, the oral bacterial community could be a useful predictor for certain production phenotypes. Future research should assess the oral microbiome’s utility in predicting health traits.

## Data Availability

The datasets presented in this study can be found in online repositories. The names of the repository/repositories and accession number(s) can be found in the article/Supplementary material.

## Supplementary material

Supplementary Figure S1 Heatmap of the differential abundance analysis at the ASV level between the first two sampling time points (50–120 and 170–240) of the oral samples. The top 80 most abundant differential abundance ASVs between 50–120 and 170–240 visualized at the genus level across oral samples.

Supplementary Figure S2 Heatmap of the differential abundance analysis at the ASV level between the first two sampling time points (50–120 and 170–240) of the rumen samples. The top 80 most abundant differential abundance ASVs between 50–120 and 170–240 visualized at the genus level across rumen samples.
