# Supplementary figures and images for: Investigating the establishment of the rumen and oral bacterial communities in beef cattle and assessing the applicability of using the oral bacterial community composition as a proxy for rumen bacterial community structure in cattle
Source: Front Microbiol. 2025 Dec 19;16:1667498. doi: 10.3389/fmicb.2025.1667498 (PMC12757392; doi:10.3389/fmicb.2025.1667498)

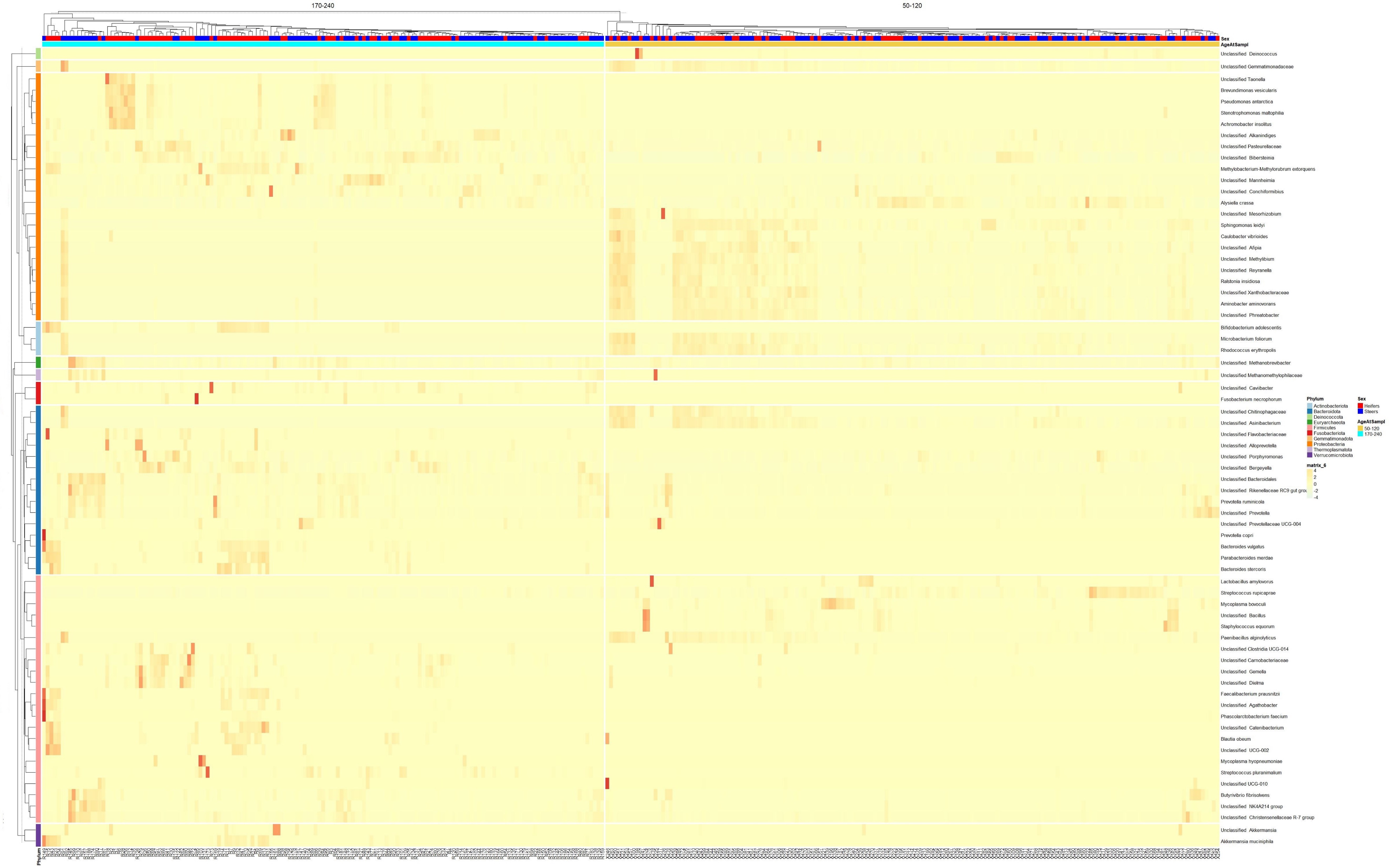

Supplement: Supplementary Figure S1 — Heatmap of the differential abundance analysis at the ASV level between the first two sampling time points (50–120 and 170–240) of the oral samples. The top 80 most abundant differential abundance ASVs between 50–120 and 170–240 visualized at the genus level across oral samples. [file Image_1.JPEG]

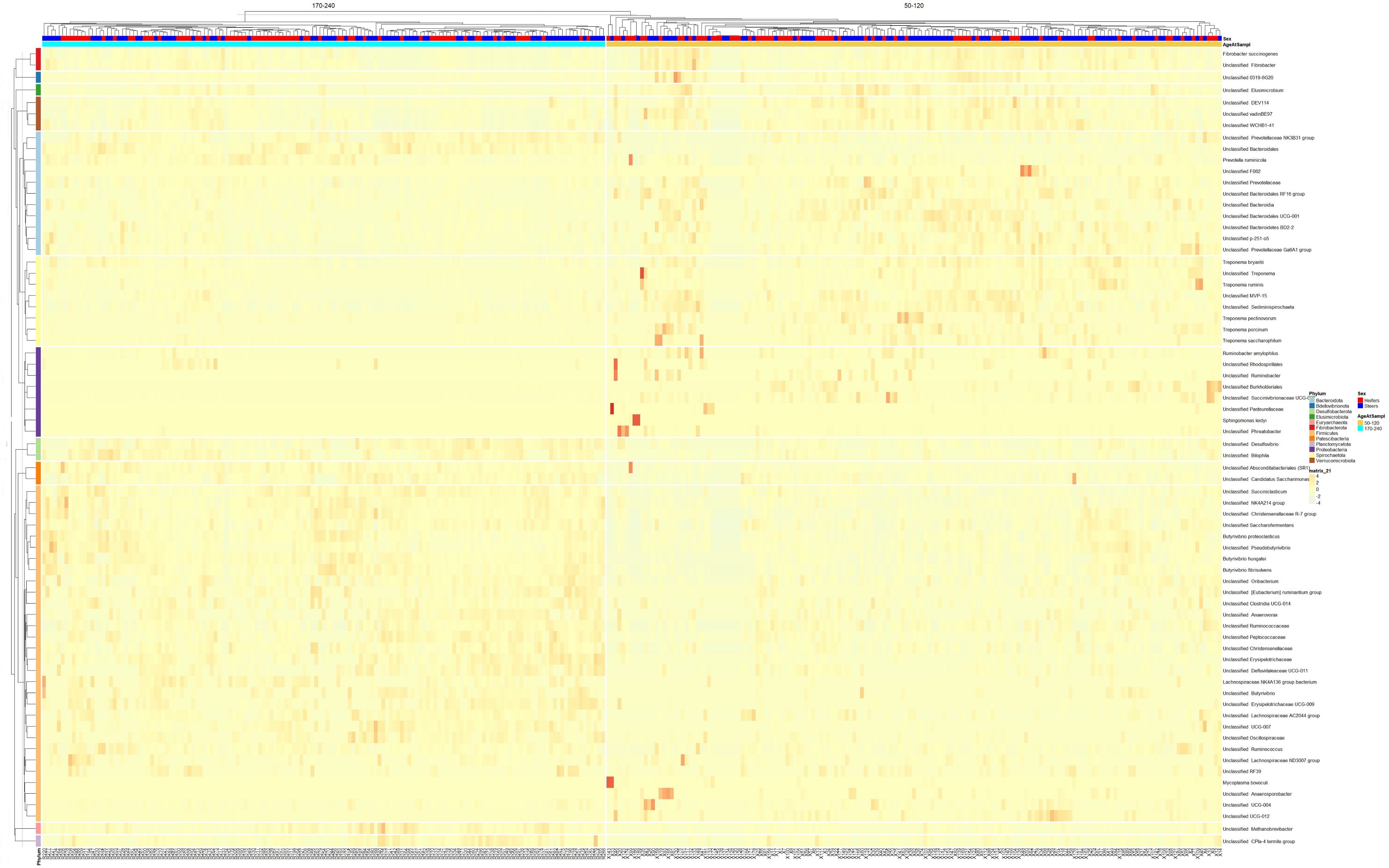

Supplement: Supplementary Figure S2 — Heatmap of the differential abundance analysis at the ASV level between the first two sampling time points (50–120 and 170–240) of the rumen samples. The top 80 most abundant differential abundance ASVs between 50–120 and 170–240 visualized at the genus level across rumen samples. [file Image_2.JPEG]
